# Supplementary material for: Effect of N-acetyl-l-cysteine on Cell Phenotype and Autophagy in Pichia pastoris Expressing Human Serum Albumin and Porcine Follicle-Stimulating Hormone Fusion Protein
Source: Molecules. 2023 Mar 29;28(7):3041. doi: 10.3390/molecules28073041 (PMC10095845; doi:10.3390/molecules28073041)
Supplement: Supplementary file 1 [file molecules-28-03041-s001.zip › molecules-2245027-supplementary.pdf]

Figure S1 Transmission electron microscopy (TEM) of the F strain treated with 5 mM NAC for 72 h. Nu: cell nucleus, Mito: mitochondria, Vac: Vacuole.

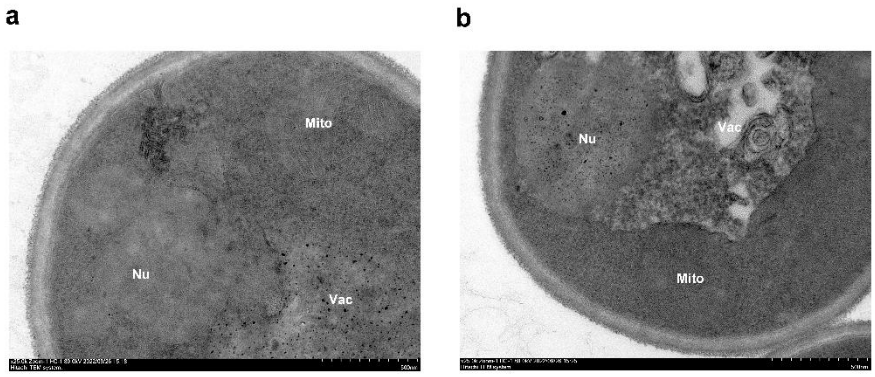

Figure S2: SDS-PAGE analysis of recombinant HSA-pFSHβ protein in the culture medium of FApe1 strain supplementing with or without NAC; +: represent the sample of culture medium supplementing with 5 mM NAC, otherwise marked -. Arrow 1 and 2 represent intact HSA-pFSHβ and truncated HSA.

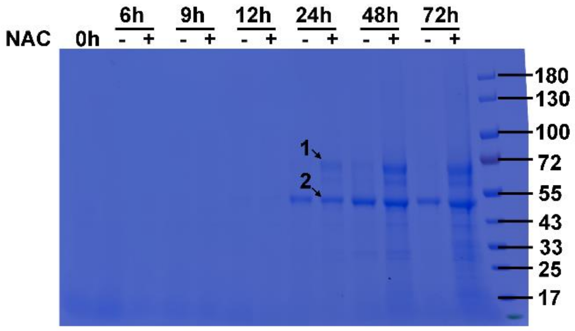

Figure S3: Analysis of HSA-pFSHβ yield in culture medium and fluorescence signal in cells. (**a**) SDS-PAGE analysis of recombinant HSA-pFSHβ protein in the culture medium of FTom20 strain supplementing with or without NAC; +: represent the sample of culture medium supplementing with 5 mM NAC, otherwise marked -.Arrow 1 and 2 represent intact HSA-pFSHβ and truncated HSA.(**b**) Total fluorescence intensity of FTom20 strain at different times of NAC treatment. The excitation and emission wavelengths are 485 nm, and 535 nm, respectively. p values were calculated using Student's t-test with $p<0.05$ considered statistically significant (Marked with *). Error bars represent means±standard deviation (SD)(n=3).

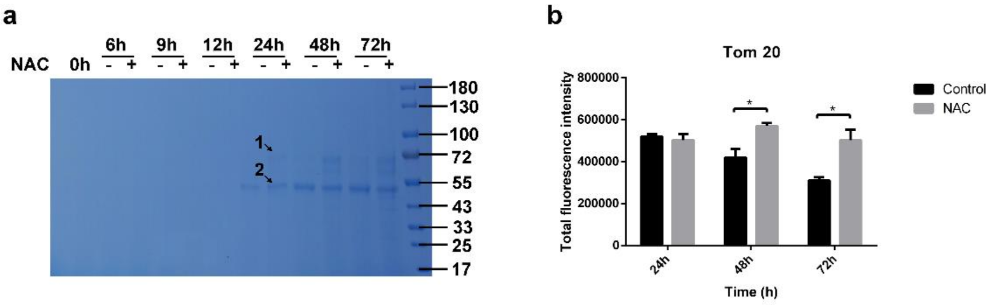

Figure S4: Apoptosis was detected using flow cytometry after NAC treatment for 72h. Cells were collected at 72 h of NAC treatment, and the ratio of apoptosis cell was analyzed using TUNEL FITC Apoptosis Detection Kit (Vazyme, Nanjing, China). Most of the cells did not undergo apoptosis after NAC treatment. Four biological replicates were performed.

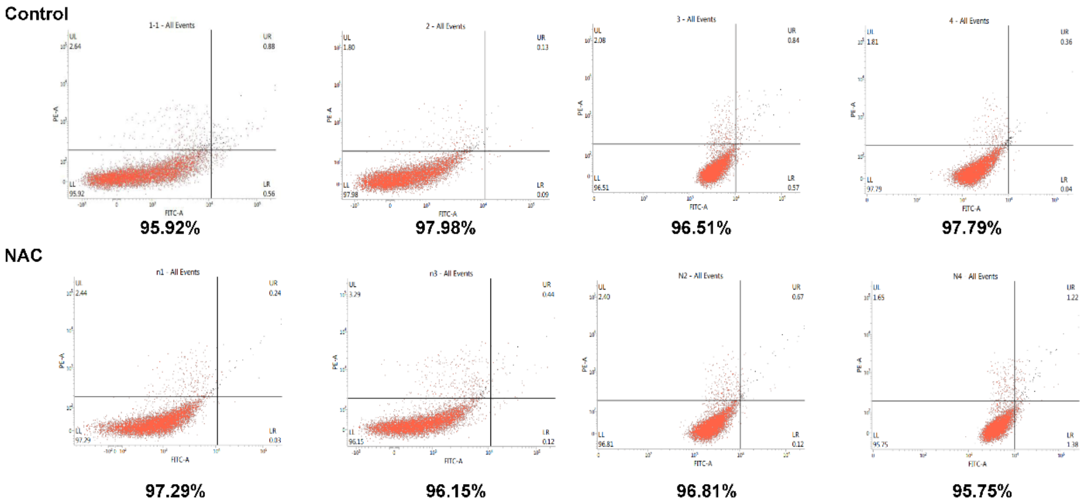

Table S1. Oligonucleotides used in this study.

Oligonucleotides	Sequence (5'-3')
YDJ-F	GCATACGAGGTTCTTTCCG
YDJ-R	TCTGTGGTCCTGTAGGTTGG
ATG1-F	TCTGTCGGTGCTGTCGTTT
ATG1-R	GTTCGTCGCTACAAGGTTGA
TRS23-F	ACAAATCGGGTGGGCTAAT
TRS23-R	GTAAAGTGCCCGCAGTGAT
GET3-F	CGGTAGTGCTGGAGGAAAC
GET3-R	CTAAAGTGGATGGCAACTGTAA
SNC2-F	ACATCAACAAAGTGGCACAAC
SNC2-R	TTAGCACCTCGTCTGAAACC
VPS21-F	TTGAGACCTCGGCAAAGAC
VPS21-R	TTGACAGGTTGATTCCGTAGA
PEX14-F	TAGGCTATCGGAGTCGTGTATC
PEX14-R	ACATTTCGGACGGCTTGAT
ARF1-F	GTGACTTGGAGGATACTGGGTT
ARF1-R	ATGAGAATACGGACTTCTTTGG
SAR1-F	GTCACGAACAAGCCAGAAGA
SAR1-R	TACCCAAGACCAAAACAGGA
ATG9-F	TCGGCAAAGTCTAAGAGCAA
ATG9-R	ATCCCCGCTAGTTGGAGAT
YPT1-F	CTGGCGGAGTGATGAAGTT
YPT1-R	ATTTGTAGAGTCCAAGGCTGAT
YKT6-F	GGTATTGCCTGCGTTTTGA
YKT6-R	CCTGAGATGGGTCCTGGTAG
SEC62-F	TCAGAGCATTATTGAGCGACA
SEC62-R	GGAAGAATCATCCCAGCCT
SEC2-F	AGATTTTCTGGGCAAGGATAG
SEC2-R	CATTTGTCTTGGTTACAGGTCC
ATG11-F	TAGCCTCCTCCACCAATCA
ATG11-R	ACCATCGTAAAACCTCCTGTC
COG4-F	AACGAGTCAAGGAACAAATACC
COG4-R	GTACCGTGGCTCATAATGCT
SEC4-F	TCAACCCTTCCTTCATCACTAC
SEC4-R	TGGACTCCTCGTCATCACATT
SRP9-F	CCTCAAAGCAAGCACAACG
SRP9-R	GTGGCTCCCCTATGAAATG
Actin-F	CCAATGAACCCAAAGTCCAA
Actin-R	CCGTCACCAGAGTCCAAAAC
ATG30-F	ATGAAGACAGCAACGGAAGC
ATG30-R	TGAAAGGGTCAGGATTGAACTA
ATG32-F	CAAATATGGAGCTGGATTTCTGTGG

ATG32-R	CTTTTCACGAGACTGGCTCATTTGT
ATG17-F	AGACGAGATTAAGGAAAGACACG
ATG17-R	CAAGAAACTGAGCAAAGCACC
ATG13-F	AAGAGGGGAACTGGAAAATAGG
ATG13-R	GCTCAAATGGGAATAATAGCACTG
ATG2-F	TGAAACTCCTTTGGATGCTC
ATG2-R	TCCGAATACACTTTTGGTCTG
PEX3-F	CTCGGCAAACTCATTTATCTTC
PEX3-R	GGCTGCTCAACAAGGGATG
yEGFP3-F	GTCGAATTCATGTCGAAGGGAGAA
yEGFP3-R	GCCTCGAGTTTGTAGAGTTCATCCATACCG
ATG8-F	TGCTCGAGCGATCGCAATTTAAAGACG
ATG8-R	GTGTCGACTTCAATCTCCTCAACACCTG
BFP-SKL-F2	GAGAATTCATGCATCATCACCACCACCACAT
BFP-SKL-R2	CCGTCGACTTACAACTTAGAGTTCAACTTATGACC
Tom20-fwd	TTTTGGTCATGCATGAGATCTTTTTTGTGGGTACGGAAAGAAAT
Tom20-rev	CCTTCGACATCTCGACATCATCGCCGTGA
yEGFP3-tom20-fwd	TGATGTCGAGATGTCGAAGGGAGAAGAGCT
pACT1-fwd	TTTTGGTCATGCATGAGATCTTCGCTGGTAATCCCGGCTTTT
pACT1-rev	GGTATCTGTCATTGTATTGATGAATTTCTTTTACTAAACTGTTTC
Ape1-fwd	ATTCATCAATACAATGACAGATACCAAGGAGTTAGC
Ape1-rev	CTCCCTTCGACATAAACTCTTCAATGCCGTCG
yEGFP3-ape1-fwd	ATTGAAGAGTTTATGTCGAAGGGAGAAGAGCT
yEGFP3-rev	ATGATGATGATGATGGTCGACTTTGTAGAGTTCATCCATACCG
1-ATG30-sgRNA-fw1	TGAAGACGCCATGTAGTAACTGATGAGTCCGTGAGGACGAAACGAGTAAGCTCGTCTTAC
2-ATG30-sgRNA-fw1	AAACGAGTAAGCTCGTCTTACTATTACAATTACAACGGTTTTAGAGCTAGAAATAGCAAG
1-ATG32-sgRNA-fw1	TGAAGACGCCATGCTTCGTCTGATGAGTCCGTGAGGACGAAACGAGTAAGCTCGTCACGA
2-ATG32-sgRNA-fw1	AAACGAGTAAGCTCGTCACGAAGACGATATAGAAGAGGTTTTAGAGCTAGAAATAGCAAG
A-sgRNA-struc-rev	CGCCATGCCGAAGCATGTTGCCCAGCCGGCGCCAGCGAGGAGGCTGGGACCATGCCGGCC
B-sgRNA-struc-rev	AGAAGACGCAAGCAGTCCAAAGCTGTCCCATTCGCCATGCCGAAGCATGTTGCCCAGCCG
C-sgRNA-struc-rev	AGGCTGGGACCATGCCGGCCAAAAGCACCGACTCGGTGCCACTTTTTCAAGTTGATAACG
D-sgRNA-struc-fw	GTTTTAGAGCTAGAAATAGCAAGTTAAAATAAGGCTAGTCCGTTATCAACTTGAAAAAGT
ATG30-R	CCTTCGACATTAAAATCTCCTGTTTGAGCTTTG
gap-ATG30-F	CCGAATTCATGTTTTCCAGAAAGCAAGTAC

ATG32-EcoRI-F	GCCGAATTCATGAAGCAAACGTATTACGAT
ATG32-yEGFP3-R	CCTTCGACATCTATACAGTGCAGCGCATCC
